# Neoadjuvant chemotherapy combined with antiangiogenic therapy and immune checkpoint inhibitors for the treatment of locally advanced gastric cancer: a real - world retrospective cohort study

**DOI:** 10.3389/fimmu.2025.1518217

**Published:** 2025-02-04

**Authors:** Zhouwei Zhan, Bijuan Chen, Shaohua Xu, Ruyu Lin, Haiting Chen, Xiaohuan Ma, Xuanping Lin, Wanting Huang, Changhua Zhuo, Yu Chen, Zengqing Guo

**Affiliations:** ^1^ Department of Medical Oncology, Clinical Oncology School of Fujian Medical University, Fujian Cancer Hospital, Fuzhou, Fujian, China; ^2^ Department of Radiation Oncology, Clinical Oncology School of Fujian Medical University, Fujian Cancer Hospital, Fuzhou, Fujian, China; ^3^ Department of Hepatobiliary and Pancreatic Surgery, Clinical Oncology School of Fujian Medical University, Fujian Cancer Hospital, Fuzhou, Fujian, China; ^4^ Clinical Oncology School of Fujian Medical University, Fujian Cancer Hospital, Fuzhou, Fujian, China; ^5^ Department of Gastrointestinal Surgical Oncology, Clinical Oncology School of Fujian Medical University, Fujian Cancer Hospital, Fuzhou, Fujian, China

**Keywords:** gastric cancer, neoadjuvant treatment, immune checkpoint inhibitors, antiangiogenesis, chemotherapy, survival

## Abstract

**Background:**

Although immune checkpoint inhibitors (ICIs) and anti-angiogenic drugs have demonstrated effectiveness in treating advanced gastric cancer (GC), their role in neoadjuvant or conversion therapy remains uncertain. This study aimed to evaluate the efficacy and safety of combining neoadjuvant chemotherapy with anti-angiogenesis and ICIs in patients with locally advanced GC (LAGC).

**Methods:**

In this cohort study, we reviewed our prospectively maintained GC database and included individuals diagnosed with clinical stage II-III GC who received neoadjuvant therapy followed by surgery between January 2022 and August 2023. The treatment protocol combined ICIs, anti-angiogenic therapy (specifically apatinib), and chemotherapy (S-1 with oxaliplatin). A systematic approach was used to document patients’ clinical and pathological characteristics, pathological findings, and survival outcomes, which were subsequently analyzed in detail.

**Results:**

A total of 38 individuals met the study’s inclusion criteria, with the majority (32 patients, 84.2%) having clinical stage III GC. All participants underwent surgery, resulting in a notable R0 resection rate of 97.4%. The rates of major pathological response (MPR) and pathological complete response (pCR) were 47.4% and 23.7%, respectively. Post-surgery, 36 patients (92.1%) received adjuvant chemotherapy. With a median follow-up of 22 months, ten patients experienced disease recurrence, including three who died from tumor relapse. The 1-year overall survival (OS) rate stood at 100%, and the disease-free survival (DFS) rate was 94.7%, with median OS and DFS yet to be reached. The neoadjuvant therapy regimen was generally well-tolerated, with no grade 5 treatment-related adverse events (TRAEs) reported. Only one patient experienced a grade 4 TRAE (immune-related hepatitis), while the most common grade 3 TRAEs included thrombocytopenia, elevated aminotransferase levels, and neutropenia.

**Conclusions:**

The combination of neoadjuvant chemotherapy, anti-angiogenic therapy, and ICIs has proven effective in treating LAGC patients, achieving high pCR rates and favorable survival outcomes while maintaining an acceptable safety profile.

## Introduction

Gastric cancer (GC) remains a major global health challenge, ranking fifth in terms of both incidence and mortality worldwide, with over 968,000 new cases and close to 660,000 deaths reported in 2022 (1). The burden of GC is particularly pronounced in Eastern Asia, where incidence rates are the highest globally, exemplified by Mongolia, which leads in incidence for both sexes (1). In contrast, regions such as sub-Saharan Africa report the lowest incidence rates, reflecting significant geographic variability in GC epidemiology. This disparity underscores the influence of genetic, environmental, and dietary factors on GC prevalence (1).

Treatment approaches for GC also vary across regions, influenced by differences in healthcare resources, cultural practices, and diagnostic advancements. In Western countries, perioperative chemotherapy is the standard of care for locally advanced GC (LAGC), supported by pivotal trials such as MAGIC and FLOT4, which demonstrated improved 5-year overall survival (OS) rates compared to surgery alone (2, 3). For example, the FLOT4 trial reported a 5-year OS rate of 45% with perioperative chemotherapy versus 36% with previous regimens. Additionally, ongoing trials such as KEYNOTE-585 and MATTERHORN are exploring the integration of immune checkpoint inhibitors (ICIs) with perioperative chemotherapy to enhance survival outcomes, particularly for patients with high PD-L1 expression (4). In contrast, East Asian countries such as Japan and South Korea prioritize early detection through nationwide screening programs, resulting in a higher proportion of early-stage diagnoses. For LAGC, the standard treatment includes D2 gastrectomy followed by adjuvant chemotherapy. Landmark trials like ACTS-GC and CLASSIC have established the efficacy of S-1 monotherapy and capecitabine plus oxaliplatin in improving survival, achieving 5-year disease-free survival (DFS) rates exceeding 70% (5, 6). Despite these advancements, perioperative chemotherapy and chemoradiotherapy are less commonly used in this region, reflecting differences in clinical practices and patient populations.

Despite significant progress, challenges remain. Approximately 40% of patients undergoing neoadjuvant chemotherapy experience recurrence or metastasis within three years post-surgery (7, 8). Recent advances in immunotherapy have brought new hope for GC treatment. ICIs, such as nivolumab and pembrolizumab (9, 10), have shown promising results in advanced GC and are now being investigated in the neoadjuvant and adjuvant settings. The neoadjuvant setting, in theory, provides an optimal environment for immunotherapy, characterized by an intact immune system, abundant neoantigens, and lower tumor clonal diversity (11). The KEYNOTE-585 trial demonstrated limited benefits of adding pembrolizumab to standard neoadjuvant therapy in untreated LAGC (4). These findings underscore the urgent need for novel multimodal strategies to address persistent gaps in GC management and improve long-term outcomes globally.

The pivotal role of tumor angiogenesis in cancer progression is well-established. Like ICIs, antiangiogenic agents target components of the tumor microenvironment (TME) beyond the tumor cells themselves. These agents can enhance the effectiveness of ICIs by promoting the infiltration and activation of CD8+ T lymphocytes (12, 13). Notably, ramucirumab, an antibody targeting VEGFR2 (14), and apatinib, a VEGFR2 tyrosine kinase inhibitor (TKIs) (15), have shown survival benefits in advanced GC. As a result, they have been approved for use in second-line and third-line treatments, respectively. These therapies have demonstrated the ability to reprogram the TME, shifting it from an immunosuppressive state to an inflamed phenotype, thereby enhancing the efficacy of ICIs in phase I/II studies (16–18). In this context, incorporating antiangiogenic agents into regimens that combine ICIs and chemotherapy offers a promising strategy to improve neoadjuvant outcomes for patients with LAGC. In this retrospective study, we aim to assess the efficacy and safety of combining neoadjuvant chemotherapy with antiangiogenic therapy (specifically apatinib) and ICIs in patients with LAGC, offering insights into the potential benefits and risks of this treatment approach.

## Materials and methods

### Patient selection

A retrospective cohort study was conducted at Fujian Cancer Hospital involving 167 treatment-naïve GC patients who received neoadjuvant therapy. Among these, 38 (22.8%) patients met the inclusion and exclusion criteria and received neoadjuvant chemotherapy combined with antiangiogenic therapy (apatinib) and ICIs between January 2022 and August 2023. The combination treatment was chosen for patients with large local tumors or extensive regional lymph node involvement, where clinical evaluation indicated that surgery would be difficult or R0 resection could not be achieved. After multidisciplinary team (MDT) discussion, neoadjuvant immune-targeted therapy combined with chemotherapy was selected to improve tumor resectability and enhance treatment efficacy.

**Figure 1:**
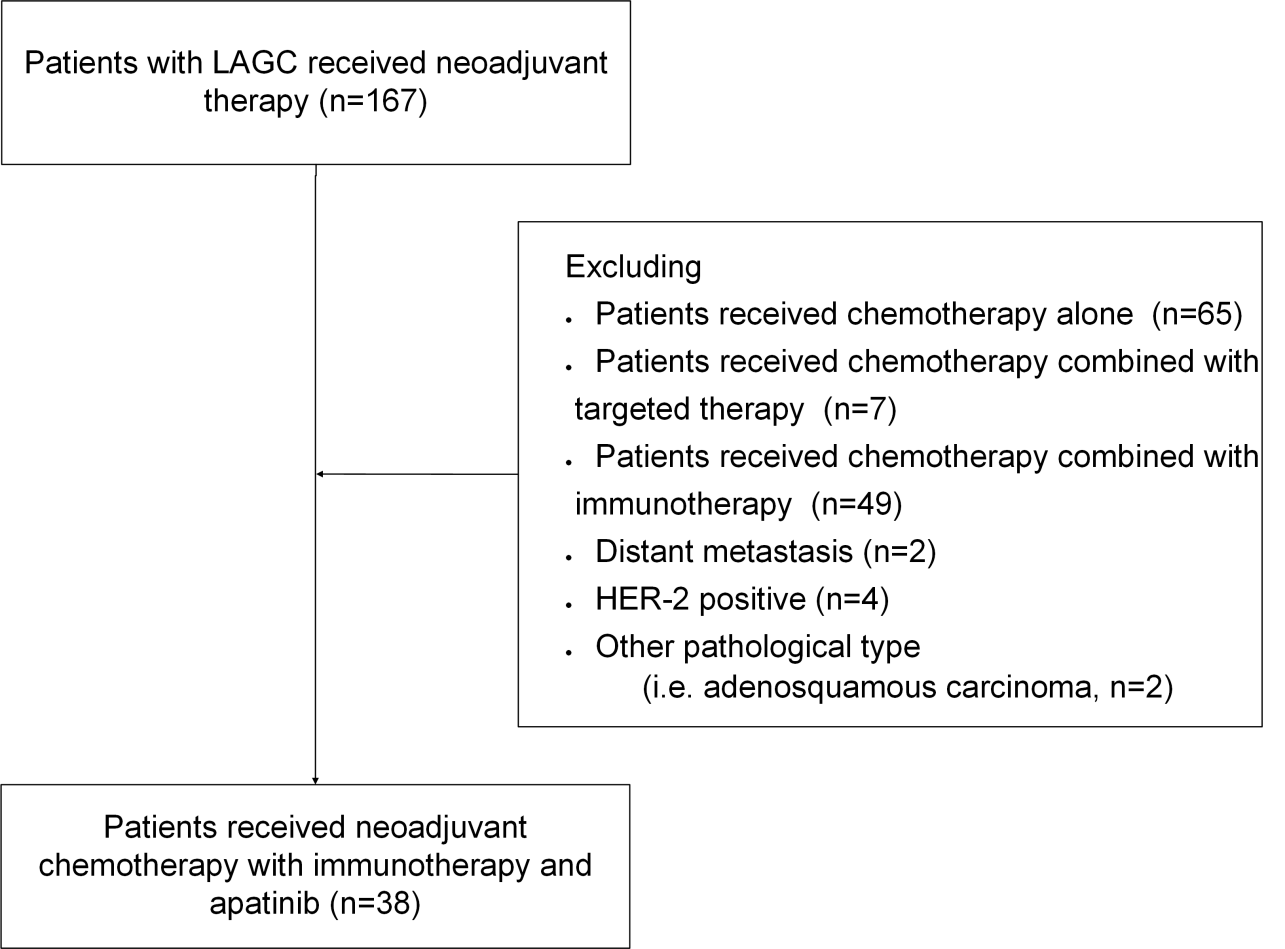
Flow chart of patient inclusion and exclusion.

The inclusion criteria for this study were as follows: histologically confirmed diagnosis of gastric adenocarcinoma, clinical stage T3-4aN0-3M0 based on the eighth edition of the American Joint Committee on Cancer (AJCC) Gastric Cancer Staging system (19, 20), age 18 or older, ECOG performance status of 0 or 1, and receipt of at least two cycles of PD-1 inhibitor therapy combined with chemotherapy and antiangiogenic therapy. Patients were excluded if they had undergone prior antitumor treatments before neoadjuvant therapy, had concurrent significant malignant tumors, impaired organ function, HER-2 positive, or distant metastasis at the time of enrollment. The case selection process is illustrated in **Figure 1**.

The study adhered to the STROCSS reporting guidelines (21) and followed the ethical principles outlined in the 1995 Declaration of Helsinki. The protocol received approval from the Fujian Cancer Hospital Ethics Committee (approval number K2024-076-01). Written informed consent was obtained from all participants. The study is registered with the Chinese Clinical Trial Registry (ChiCTR2400081423). This rigorous protocol ensured the study’s ethical integrity and scientific validity throughout the retrospective analysis at Fujian Cancer Hospital.

### Treatment protocols

Chemotherapy in this study involved two to six cycles of the standardized SOX regimen. For each SOX cycle, patients received a 2-hour intravenous infusion of oxaliplatin at a dose of 130 mg/m² on day one, combined with oral S-1. The dosage of S-1 was adjusted according to the patient’s body surface area (BSA): 40 mg twice daily for those with a BSA below 1.25 m², 50 mg twice daily for BSA between 1.25-1.5 m², and 60 mg twice daily for those with a BSA above 1.5 m². Apatinib was administered orally at 250 mg daily, while nivolumab, camrelizumab, and tislelizumab, all PD-1 inhibitors, were administered intravenously at 200 mg per cycle. This regimen was administered for 2 weeks, followed by a 1-week rest, making up a 21-day cycle. Dose adjustments (such as interruptions, delays, or reductions) were allowed in cases of grade ≥3 hematologic or grade ≥2 nonhematologic adverse events (AEs). Treatment could be discontinued if the patient refused, the tumor progressed, toxicity became intolerable, or the investigator decided to stop.

After completing the final cycle of neoadjuvant therapy, patients underwent a standardized gastrectomy with D2 lymphadenectomy, performed within 2 to 4 weeks. The surgical approach was tailored according to the tumor’s location and size, following the guidelines set by the Japanese Research Society for the Study of GC (22), ensuring sufficient resection margins. Comprehensive surgical data, including the surgical approach, operative time, blood loss, length of postoperative hospital stay, and resection margins, were carefully extracted from electronic medical records. Postoperative complications occurring within 30 days of surgery were systematically classified using the Clavien-Dindo classification (23). Following surgery, patients began an adjuvant treatment course that mirrored the chemotherapy regimen used during the neoadjuvant phase, starting around four weeks after the procedure. Postoperative follow-up included regular CT scans every three months during the first year and every six months thereafter.

### Outcome assessment

The primary outcome measures focused on pathological responses, including the rates of pathological complete response (pCR) and major pathological response (MPR), as well as radiological responses. Radiological tumor responses were evaluated using the Response Evaluation Criteria in Solid Tumors (RECIST) version 1.1, categorizing outcomes into complete response (CR), partial response (PR), stable disease (SD), and progressive disease (PD) (24). These assessments were conducted at baseline and pre-surgery. The objective response rate (ORR) was defined as the highest overall response of CR or PR achieved, while the disease control rate (DCR) encompassed CR, PR, or SD. Postoperative pathological responses of the primary tumor were evaluated using the Becker criteria for Tumor Regression Grade (TRG), which categorizes responses into TRG1a (no remaining tumor cells), TRG1b (less than 10% remaining tumor cells), TRG2 (10–50% remaining tumor cells), and TRG3 (more than 50% remaining tumor cells). pCR was defined as TRG1a, and MPR included both TRG1a and TRG1b (25). The cTNM and ypTNM staging systems adhered to the 8th edition of the AJCC staging guidelines. Additional outcome measures included OS, defined as the time from the start of neoadjuvant treatment to death from any cause, and DFS, defined as the time from the initiation of neoadjuvant treatment to either disease recurrence or death from any cause. Additionally, treatment-related adverse events (TRAEs) were evaluated according to the Common Terminology Criteria for Adverse Events (CTCAE) version 5.0. HER2 positivity was assessed using immunohistochemical staining.

### Statistical analyses

All statistical analyses were performed using SPSS version 24.0 (IBM SPSS Inc., Chicago, IL, USA) and GraphPad Prism 9.0 (GraphPad Software Inc., San Diego, CA, USA). Continuous variables were expressed as mean ± standard deviation (SD) or median with range, depending on data distribution, while categorical variables were summarized as frequencies and percentages. Kaplan-Meier survival analysis was employed to estimate OS and DFS, with differences assessed using the log-rank test. Univariate and multivariate Cox regression analyses were conducted to evaluate potential prognostic factors for DFS. A two-tailed *P*-value of <0.05 was considered statistically significant.

## Results

This study included 38 patients who met the eligibility criteria and completed the entire course of neoadjuvant chemotherapy combined with antiangiogenic therapy and ICIs. These patients underwent a median of four cycles of neoadjuvant immunochemotherapy (range: 2 to 6 cycles) before surgery. The average interval between the completion of neoadjuvant therapy and surgery was 26 days, ranging from 14 to 39 days. The baseline characteristics of the patients are summarized in **Table 1**. The median age was 65 years, with a range of 32 to 73 years. Most patients were male, comprising 30 (78.9%) of the cohort. Regarding ECOG performance status, 31 patients (81.6%) had a score of 0, while 7 (18.4%) had a score of 1. Clinically, 6 patients (15.8%) were diagnosed with TNM stage II, while 32 (84.2%) had stage III disease. Adjuvant chemotherapy was administered to 35 patients (92.1%).

**Table 1:** Baseline characteristics of patients.

Characteristic	Patients (n = 38)
Gender, n (%)	
Male	30 (78.9%)
Female	8 (21.1%)
Age (years), n (%)	
<60	14 (36.8%)
>60	24 (63.2%)
ECOG PS, n (%)	
0	31 (81.6%)
1	7 (18.4%)
Tumor location, n (%)	
Gastric	30 (78.9%)
Gastroesophageal junction	8 (21.1%)
Tumor size (cm), n (%)	
<3	32 (84.2%)
>=3	6 (15.8%)
Tumor differentiation, n (%)	
High-moderate	10 (26.3%)
Poor	28 (73.7%)
Lauren type, n (%)	
Intestinal	8 (21.1%)
Diffuse-mixed	30 (78.9%)
Clinical T stage, n (%)	
T3	3 (7.9%)
T4	35 (92.1%)
Clinical N stage, n (%)	
0	4 (10.5%)
+	34 (89.5%)
Clinical TNM stage, n (%)	
II	6 (15.8%)
III	32 (84.2%)
Neoadjuvant cycles, median (range)	4 (2-6)
Immunotherapeutic drugs, n (%)	
Nivolumab	1 (2.6%)
Camrelizumab	3 (7.9%)
Tislelizumab	34 (89.5%)
Adjuvant therapy, n (%)	
Yes	35 (92.1%)
No	3 (7.9%)

### Tumor response and survival outcomes

Following neoadjuvant therapy, all patients underwent standardized D2 surgical resection, with detailed surgical data provided in **Table 2**. The median surgery duration was 330 minutes, ranging from 150 to 476 minutes, and median blood loss was 50 mL, with a range of 20 to 1000 mL. The median postoperative hospital stay was 12 days. Only one patient required an R1 resection, achieving an R0 resection rate of 97.4%. Postoperative complications were observed in 8 patients (21.1%), mainly including pulmonary infections in 5 patients (13.2%), anastomotic leakage in 2 patients (5.3%), and abdominal infections in 3 patients (7.9%). Most of these complications were manageable, falling under Clavien-Dindo grades I and II, with no patients experiencing grade III complications.

**Table 2:** Surgery information.

Characteristic	Patients (n=38)
Surgical technology, n (%)	
Open	1 (2.6%)
Laparoscopic	37 (97.4%)
Gastrectomy type, n (%)	
Subtotal	11 (28.9%)
Total	27 (71.0%)
Operative time (min), mean ± SD	330 (250, 380)
Blood loss (mL), median (IQR)	50 (30, 80)
Postoperative hospital stays (days), median (IQR)	12 (10, 17)
R0 resection rate, n (%)	38 (97.4%)
Harvested lymph nodes, median (range)	44 (18-76)
Postoperative complications, overall, n (%)	8 (21.1%)
Pulmonary infection	5 (13.2%)
Anastomotic leakage	2 (5.3%)
Abdominal infections	3 (7.9%)
Clavien-dindo classification, n (%)	
Grade I	0 (0%)
Grade II	8 (21.1%)
Grade III	0 (0%)
Vascular invasion, n (%)	
Positive	18 (47.4%)
Negative	20 (52.6%)
Neural invasion, n (%)	
Positive	12 (31.6%)
Negative	26 (68.4%)

**Figure 2:**
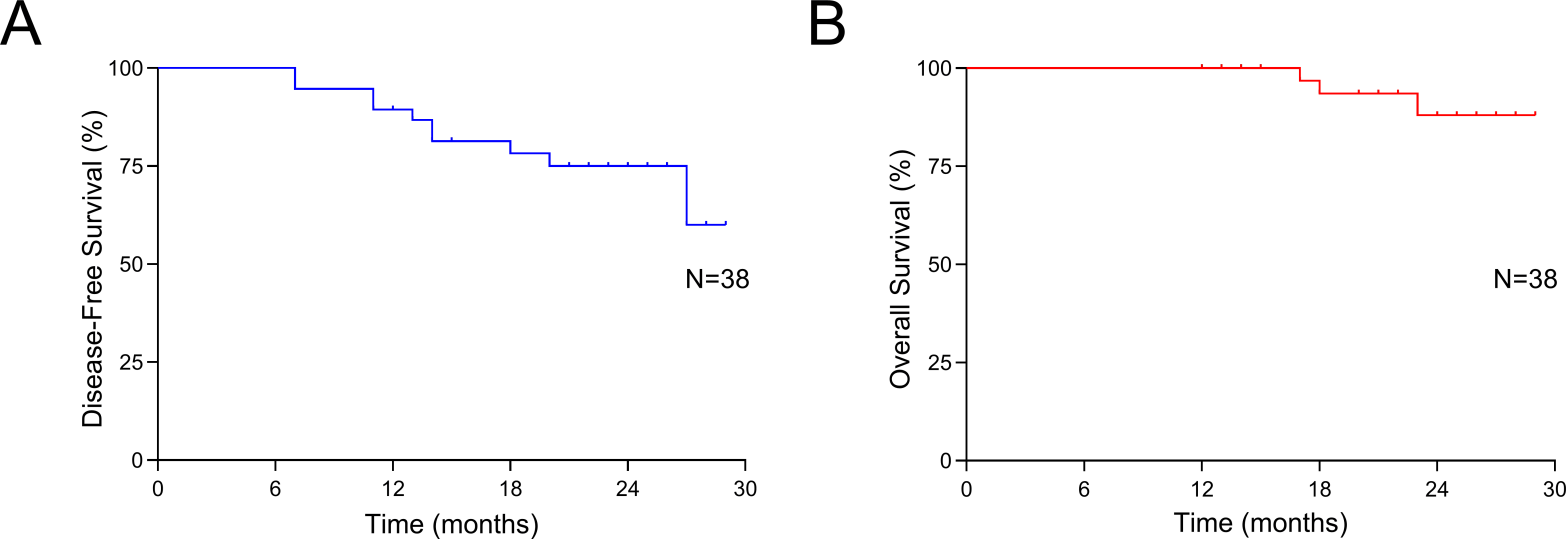
Survival outcomes. **(A)** Disease-free survival of all patients; **(B)** Overall survival of all patients.

As shown in **Table 3**, among the 38 patients who received neoadjuvant chemotherapy combined with antiangiogenesis and ICIs, 24 (63.2%) achieved a radiological PR, while 14 (36.8%) had SD. No cases of CR or PD were recorded. The ORR and DCR were 63.2% and 100.0%, respectively. Final pathological assessments revealed the following TRG distribution: TRG1a in 9 patients (23.7%), TRG1b in 9 (23.7%), TRG2 in 14 (36.8%), and TRG3 in 6 (15.8%). The rates of MPR and pCR were 47.4% (18/38) and 23.7% (9/38), respectively. As of the data cutoff on February 20th, 2024, the median follow-up duration was 15 months. Six patients had died due to tumor recurrence, while four were alive with recurrent disease. The median OS and DFS had not yet been reached. The 1-year OS and DFS rates were 100% and 94.7%, respectively, as shown in **Figure 2**. The results of **Supplementary Table 1** show that none of the examined baseline factors, including age, gender, tumor location, size, differentiation, clinical staging, neoadjuvant cycles, and immunotherapeutic drugs, were significantly associated with DFS in both univariate and multivariate Cox regression analyses. This indicates the lack of a single dominant prognostic factor affecting DFS in the studied cohort​.

**Table 3:** Radiological and pathological responses.

Characteristic	Patients (n = 38)
Radiologic responses, n (%)	
CR	0 (0%)
PR	24 (63.2%)
SD	14 (36.8%)
PD	0 (0%)
ORR, n (%)	24 (63.2%)
DCR, n (%)	38 (100.0%)
Pathological responses, n (%)	
TRG 1a	9 (23.7%)
TRG 1b	9 (23.7%)
TRG 2	14 (36.8%)
TRG 3	6 (15.8%)
CPR, n (%)	9 (23.7%)
MPR, n (%)	18 (47.4%)
ypT stage, n (%)	
T0	9 (23.7%)
T1	6 (15.8%)
T2	4 (10.5%)
T3	12 (31.6%)
T4a	7 (18.4%)
ypN stage, n (%)	
N0	22 (57.9%)
N1	9 (23.7%)
N2	2 (5.3%)
N3	5 (13.2%)
ypTNM stage, n (%)	
0	9 (23.7%)
I	8 (21.1%)
II	13 (34.2%)
III	8 (21.1%)

CR complete response, PR partial Response, SD stable disease, PD progressive disease, ORR objective response rate, DCR disease control rate, TRG tumor regression grade, MPR major pathological response rate, CPR complete response rate.

### Safety

**Table 4** details the TRAEs observed during neoadjuvant therapy. Among the 38 patients, 36 (94.7%) experienced at least one adverse event. The most common TRAEs, affecting over 10% of patients, included thrombocytopenia (55.3%, 21 patients), neutropenia (44.7%, 17 patients), increased aspartate aminotransferase (44.7%, 17 patients), leukopenia (42.1%, 16 patients), elevated alanine aminotransferase (42.1%, 16 patients), anemia (31.6%, 12 patients), nausea (39.5%, 15 patients), diarrhea (34.2%, 13 patients), and anorexia (23.7%, 9 patients). Most of the TRAEs were mild, classified as grade 1 or 2. Only 7 patients (18.4%) experienced grade 3-4 TRAEs, including increased aspartate aminotransferase (3 patients, 7.9%), elevated alanine aminotransferase (2 patients, 5.3%), elevated bilirubin (2 patients, 5.3%), neutropenia (2 patients, 5.3%), anemia (1 patient, 2.6%), and thrombocytopenia (1 patient, 2.6%). Notably, no grade 5 TRAEs were observed during the neoadjuvant treatment.

**Table 4:** Summary of treatment-related adverse events during neoadjuvant treatment (n = 38).

Adverse events	Any grade	Grade 3	Grade 4
Haematological toxicity, (n, %)			
Anemia	12 (31.6%)	1 (2.6%)	0 (0%)
Leukopenia	16 (42.1%)	0 (0%)	0 (0%)
Neutropenia	17 (44.7%)	2 (5.3%)	0 (0%)
Thrombocytopenia	21 (55.3%)	1 (2.6%)	0 (0%)
Gastrointestinal toxicity, (n, %)			
Nausea	15 (39.5%)	0 (0%)	0 (0%)
Vomiting	8 (21.1%)	0 (0%)	0 (0%)
Anorexia	9 (23.7%)	0 (0%)	0 (0%)
Diarrhea	13 (34.2%)	0 (0%)	0 (0%)
Bleeding	7 (17.4%)	0 (0%)	0 (0%)
Hepatotoxicity, (n, %)			
AST elevation	17 (44.7%)	2 (5.3%)	1 (2.6%)
ALT elevation	16 (42.1%)	1 (2.6%)	1 (2.6%)
Bilirubin increased	6 (15.8%)	1 (2.6%)	0 (0%)
Cardiac toxicity, (n, %)			
Hypertension	6 (15.8%)	0 (0%)	0 (0%)
Renal toxicity			
Proteinuria	12 (31.6%)	0 (0%)	0 (0%)
Dermal toxicity			
Hand-foot syndrome	8 (21.1%)	0 (0%)	0 (0%)
Immunotherapy-related adverse events, (n, %)			
Elevated creatine kinase	2 (5.3%)	0 (0%)	0 (0%)
Immune pneumonitis	2 (5.3%)	0 (0%)	0 (0%)
Immune-related hepatitis	0 (0%)	0 (0%)	1 (2.6%)
Hypothyroidism	3 (7.9%)	0 (0%)	0 (0%)
Hyperthyroidism	1 (2.6%)	0 (0%)	0 (0%)

## Discussion

This study provides strong evidence supporting the benefits of combining chemotherapy with ICIs and antiangiogenic agents as a neoadjuvant therapy for patients with LAGC. The results are noteworthy, achieving a pCR rate of 23.7%, an MPR rate of 47.4%, and a remarkably high R0 resection rate of 97.4%. Additionally, this innovative neoadjuvant approach has demonstrated a favorable safety profile, without causing delays in performing radical surgery. The survival outcomes are encouraging as well, with a one-year OS rate of 100% and a one-year DFS rate of 94.7%.

Historically, LAGC has been linked to a poor prognosis, particularly among patients in stages IIIA, IIIB, and IIIC, where five-year survival rates are approximately 30.5%, 20.1%, and 8.3%, respectively (26). However, recent advancements, including the pivotal findings of the MAGIC trial, have established the role of perioperative chemotherapy in the treatment of LAGC (2). Today, neoadjuvant chemotherapy has become a standard approach to increase R0 resection rates and improve DFS (27). Common chemotherapy regimens for the perioperative treatment of GC include CapeOX, SOX, and FLOT. However, the effectiveness of these regimens is often constrained by low rates of pathological regression (28). Previous studies have reported suboptimal pCR rates of 4% to 9% with neoadjuvant CapeOX therapy (29–31). In contrast, the FLOT regimen has demonstrated superior results in the neoadjuvant setting, achieving a pCR rate of 16% and an MPR rate of 37%, surpassing both CapeOX and SOX regimens (3). To further enhance these outcomes, researchers are actively investigating strategies to intensify neoadjuvant treatment regimens, with the goal of improving the prognosis for patients with LAGC.

Recent studies strongly support the use of anti-PD-1/PD-L1 therapies in this patient population, with a primary focus on pCR as a key outcome measure. For example, the KEYNOTE-585 trial demonstrated a significant 10.9% increase in pCR rates when pembrolizumab was added to chemotherapy, compared to placebo plus chemotherapy. This combination also resulted in an extension of median event-free survival (4). Similarly, the addition of camrelizumab to the FLOT regimen led to enhanced pathological regression rates (15%) and a perfect R0 resection rate (100%), significantly surpassing the FLOT-only group, which achieved rates of 5% and 90.5%, respectively (32). Other combinations, including camrelizumab with FOLFOX (33), camrelizumab with SOX/CapeOX (34), sintilimab with CapeOX (35), and tislelizumab with SOX (36), have also proven effective as neoadjuvant regimens for LAGC patients. These therapies have demonstrated promising pCR rates of 8%, 24.1%, 19.4%, and 25.0%, respectively, along with good tolerability. A meta-analysis further supports the safety, feasibility, and enhanced pathological response of ICI-based perioperative treatment compared to chemotherapy alone (37). In the DANTE study, patients were randomized to receive perioperative FLOT with or without atezolizumab, showing a significant increase in pCR rates for those treated with atezolizumab (24%) compared to the FLOT-only group (15%) (38). Similarly, the phase 3 MATTERHORN study demonstrated that adding an anti-PD-1 to perioperative FLOT significantly improved pCR and MPR rates, reaching 19% and 27%, respectively, compared to 7% and 14% in the FLOT-only control group (39). The ICONIC study also reported promising results, with MPR and pCR rates of 21% and 15%, respectively, for patients receiving perioperative FLOT combined with avelumab. However, the study was terminated early as it was unlikely to achieve the target pCR of 25% (40). Despite the observed benefits in terms of pCR and potential clinical improvements in median event-free survival, these gains have not yet translated into a statistically significant extension of event-free survival (4). This highlights the pressing need to develop more tolerable and effective combination therapies for this patient population. While current treatments show promise, there remains significant room for improvement, particularly in achieving better OS outcomes.

Angiogenesis, the formation of new blood vessels, is pivotal in tumor growth and metastasis, making it a hallmark of cancer (41, 42). Targeting this process, anti-angiogenic therapies have proven effective in treating various cancers, including GC, utilizing agents like anti-vascular endothelial growth factor (VEGF) antibodies and TKIs (14, 15, 43). Emerging evidence suggests that anti-angiogenic agents could be effective as neoadjuvant treatments for resectable tumors, rather than being confined to end-line options for chemo-refractory cases (44, 45). In the context of locally advanced oesophagogastric adenocarcinoma, Phase 2 results from the RAMSES trial demonstrated that adding ramucirumab, a VEGFR-2 inhibitor, to neoadjuvant FLOT therapy significantly improved R0 resection rates, even though pCR rates remained unchanged (46). Similarly, the ST03 trial found that combining bevacizumab, an anti-VEGF monoclonal antibody, with the perioperative ECX regimen did not improve OS in patients with potentially resectable oesophagogastric adenocarcinoma (47). So far, combining anti-angiogenic antibodies with neoadjuvant chemotherapy has not shown a definitive survival advantage in this patient population. Nonetheless, ongoing research is focused on optimizing the use and integration of anti-angiogenic therapies in the neoadjuvant setting, aiming to enhance outcomes for patients with resectable tumors.

Inhibiting the VEGF/VEGFR pathway has been shown to effectively disrupt angiogenesis and reduce immunosuppression within the TME, thereby enhancing the local immune response when used in combination with ICIs (13, 48). VEGF/VEGFR pathway inhibition reduces hypoxia, normalizes aberrant vasculature, and promotes immune cell trafficking, while concurrently limiting the activity of immunosuppressive cells such as myeloid-derived suppressor cells (MDSCs) and regulatory T cells (Tregs) (49). These changes create a more immunostimulatory TME, which enhances the efficacy of ICIs. ICIs further reinvigorate exhausted T cells by blocking inhibitory PD-1/PD-L1 interactions, thereby restoring cytotoxic T lymphocyte (CTL) activity and sustaining anti-tumor immunity within the TME (50). Chemotherapy also plays a vital role in this synergy by inducing immunogenic cell death (ICD) (51). ICD promotes the release of tumor antigens and danger-associated molecular patterns, which activate dendritic cells and prime tumor-specific T cells, thereby amplifying the immune response and complementing the effects of both ICIs and anti-angiogenic therapy. Preclinical studies using humanized GC-PDX models have demonstrated that apatinib can block the CXCL5/CXCR2 axis, counteracting the upregulation of CXCL5 induced by anti-PD-1 therapy in GC epithelium, and amplifying the therapeutic effects of anti-PD-1 immunotherapy (52). In GC tumor-bearing mice, the combination of a PD-1 inhibitor and apatinib significantly increased CD4+ and CD8+ T cell infiltration in the TME, while reducing MDSCs, thereby boosting the effectiveness of immunotherapy (53).

Clinical evidence also supports the synergistic potential of this combination. In a Phase II clinical trial combining ICIs with concurrent radiotherapy, patients with LAGC achieved notable outcomes, including an R0 resection rate of 95.0%, an MPR rate of 73.7%, and a pCR rate of 42.1%, even among 17.9% of cases classified as T4bN+ (54). A subsequent multicenter, randomized controlled trial evaluated neoadjuvant treatment regimens in LAGC patients, comparing anti-PD-1 immunotherapy and apatinib combined with nab-paclitaxel and S-1 (SAP) against camrelizumab plus SAP and SAP alone. The combined therapy group demonstrated significantly higher MPR and pCR rates, achieving 33.3% and 16.3%, respectively (55). Similar pathological responses (pCR: 23.7%; MPR: 47.4%) were observed, highlighting the advantages of combining neoadjuvant immunotherapy, anti-angiogenesis, and chemotherapy for treating LAGC. Beyond these encouraging pathological outcomes, the short-term survival data was also promising, with a 1-year OS rate of 100% and a 1-year DFS rate of 94.7%. However, at the time of reporting, the median OS and DFS had not yet been reached. These results indicate that the combined approach of immunotherapy, anti-angiogenesis, and chemotherapy could be a promising treatment strategy for patients with LAGC.

Safety is a crucial consideration in all treatment strategies, and our study highlights a favorable safety profile for the combined use of chemotherapy, ICIs, and antiangiogenic agents. Hematologic events were the most common TRAEs observed during neoadjuvant therapy with this combination, including some cases of severe grade 3-4 events. These findings are consistent with previous studies on combinations like sintilimab with CapeOX (35) and apatinib plus SOX (56), suggesting a reliable safety profile. Similar to earlier research, our study did not observe any cases of thromboembolism (15, 55). Our main concern was the potential impact of apatinib on post-surgical wound and anastomotic healing, given the known effects of VEGF inhibitors on anastomotic recovery. Interestingly, our study found a lower incidence of anastomotic leakage compared to the apatinib, camrelizumab, nano-particle albumin-bound (nab)-paclitaxel, and S-1 (CA-SAP) regimen reported in the Arise-FJ-G005 study (55). Thrombocytopenia, frequently observed with platinum-based therapies, is mainly attributed to the cytotoxic effects these agents have on megakaryocytes (57). Oxaliplatin, a platinum-based agent, is widely used in the treatment of GC. Thrombocytopenia caused by bone marrow suppression typically develops within a few days of oxaliplatin exposure, with platelet levels reaching their lowest point around 10 days post-treatment. Importantly, significant bleeding is rare, as the thrombocytopenia is usually mild (57). The mechanism behind oxaliplatin-induced immune-mediated thrombocytopenia is believed to involve the formation of specific antibodies targeting platelet glycoproteins. These antibodies become specific to platelet epitopes in the presence of oxaliplatin. Patients undergoing oxaliplatin-based therapy may develop multiple antibodies targeting different drugs, all of which can contribute to drug-induced immune thrombocytopenia. Oxaliplatin has been strongly linked to the occurrence of chemotherapy-induced thrombocytopenia (CIT)ally (58), immune thrombocytopenia (ITP) has been reported as a secondary effect of PD-1/PD-L1 inhibitor therapies (59). A pharmacovigilance study, complemented by a systematic review using data from the United States Food and Drug Administration’s Adverse Event Reporting System (FAERS), emphasizes the potentially life-threatening nature of ICI-induced ITP. This finding underscores the critical need for clinicians to recognize the seriousness of this adverse event (60). In our study, thrombocytopenia was observed in 55.3% of patients, with third-degree thrombocytopenia occurring in 2.6% of cases. These results suggest that the combined use of oxaliplatin and PD-1 inhibitors may increase the risk of immune thrombocytopenia. Thus, it is crucial for clinicians to remain vigilant about this potential complication.

Immunotherapy-induced hepatotoxicity can vary greatly in severity, from mild elevations in liver aminotransferase levels to, in rare instances, fulminant liver failurerted incidence of immunotherapy-induced hepatitis varies widely (61), with clinical trials typically estimating a relatively low occurrence of around 5.8% (62). In contspective studies have reported much higher rates, with some findings suggesting incidences as high as 64% (63). In the FAERS database, hepatic failure was observed in 0.19% of patients (18,454 out of 9,647,655), with 654 cases linked to checkpoint inhibitor therapy (64). In our study, two additional patients experienced CTCAE grade 3 liver injury, which we attributed to oxaliplatin treatment. Previous studies have reported cases of severe liver injury associated with the combined use of oxaliplatin and PD-1 inhibitors (65, 66). These findings highlight the critical need for vigilant monitoring of liver toxicity in patients receiving this combination therapy.

This study has several limitations that should be acknowledged. First, its retrospective design inherently introduces the possibility of selection bias, which, combined with the relatively small sample size, may limit the generalizability of our findings. Second, the single-arm nature of the study is a notable constraint, as the lack of a control group precludes direct comparisons with other established treatment regimens, making it challenging to comprehensively assess the relative efficacy of this neoadjuvant approach. Third, the short follow-up period prevents an accurate evaluation of long-term outcomes, including median OS and PFS. To address this limitation, we plan to extend the follow-up period to obtain more robust survival data and report updated results in future studies. Lastly, large-scale, prospective, randomized controlled trials are essential to validate the clinical utility of this combined regimen. Such studies would provide stronger evidence regarding the efficacy, safety, and long-term benefits of integrating chemotherapy, ICIs, and anti-angiogenic therapy in the neoadjuvant setting for patients with LAGC.

## Conclusions

In conclusion, the combination of neoadjuvant chemotherapy, ICIs, and antiangiogenic agents shows promise as an effective and feasible treatment strategy for patients with LAGC. This integrated approach demonstrated encouraging outcomes, including high pathological response rates and favorable short-term survival, suggesting its potential as a valuable neoadjuvant option. However, the study’s retrospective nature, limited sample size, and lack of a control group present challenges to the generalizability of the findings. Additionally, the relatively short follow-up period precluded a comprehensive evaluation of long-term outcomes such as median OS and PFS. To solidify the clinical significance of this regimen, further validation through larger-scale, randomized controlled trials is crucial. Such studies would provide more robust evidence regarding the long-term efficacy and safety of this combination therapy, ensuring better treatment decisions for LAGC patients.

## Data Availability

The raw data supporting the conclusions of this article will be made available by the authors, without undue reservation.
