# Supplementary material for: Neoadjuvant chemotherapy combined with antiangiogenic therapy and immune checkpoint inhibitors for the treatment of locally advanced gastric cancer: a real - world retrospective cohort study
Source: Front Immunol. 2025 Feb 4;16:1518217. doi: 10.3389/fimmu.2025.1518217 (PMC11832677; doi:10.3389/fimmu.2025.1518217)
Supplement: Supplementary file 1 [file Table1.docx]

Supplementary Table1. Prognostic factors for disease-free survival by univariate and multivariate cox regression analyses

| Variables | Univariate analyses | | | Multivariate analyses | | |
| --- | --- | --- | --- | --- | --- | --- |
|  | HR | 95%CI | *P* | HR | 95%CI | *P* |
| Gender | 1.417 | 0.284-7.068 | 0.671 | 0.001 | 0-18.145 | 0.158 |
| Age | 1.521 | 0.393-5.885 | 0.544 | 0.505 | 0.032-7.890 | 0.626 |
| ECOG PS | 0.463 | 0.087-2.472 | 0.368 | 14625.611 | 0.117-1828097857 | 0.109 |
| Tumor location | 0.929 | 0.193-4.477 | 0.927 | 1.321 | 0.048-36.486 | 0.869 |
| Tumor size | 1.890 | 0.531-6.724 | 0.326 | 0.153 | 0.005-4.577 | 0.279 |
| Tumor differentiation | 2.014 | 0.427-9.499 | 0.377 | 0.000 | 0.000-2.140 | 0.073 |
| Lauren type | 1.138 | 0.422-3.071 | 0.798 | 0.005 | 0.000-22.990 | 0.220 |
| Clinical T stage | 1.052 | 0.131-8.443 | 0.962 | 3056.915 | 0.000-1.130*10^153^ | 0.964 |
| Clinical N stage | 1.441 | 0.182-11.417 | 0.729 | 0.000 | 0.000-5.975*10^141^ | 0.962 |
| Clinical TNM stage | 1.123 | 0.238-5.294 | 0.884 | 0.026 | 0.000-8.659*10^147^ | 0.983 |
| Neoadjuvant cycles | 1.083 | 0.278-4.214 | 0.909 | 14.962 | 0.144-1557.881 | 0.254 |
| Immunotherapeutic drugs | 0.361 | 0.122-1.066 | 0.065 | 0.728 | 0.005-107.122 | 0.901 |
| Surgical technology | 21.092 | 0.000-405943905.3 | 0.722 | 1.538 | 0.000  2.092*10^107^ | 0.997 |
| Gastrectomy type | 0.947 | 0.244-3.676 | 0.937 | 0.273 | 0.029-2.569 | 0.256 |
| Adjuvant therapy | 0.956 | 0.119-7.659 | 0.966 | 5.159 | 0.036-742.037 | 0.517 |
